# Effect of *Burkholderia tropica* and *Herbaspirillum frisingense* strains on sorghum growth is plant genotype dependent

**DOI:** 10.7717/peerj.5346

**Published:** 2018-07-24

**Authors:** Thiago R. Schlemper, Maurício R. Dimitrov, Federico A.O. Silva Gutierrez, Johannes A. van Veen, Adriana P.D. Silveira, Eiko E. Kuramae

**Affiliations:** 1 Department of Microbial Ecology, Netherlands Institute of Ecology (NIOO-KNAW), Wageningen, The Netherlands; 2 Leiden University, Institute of Biology, Leiden, The Netherlands; 3 Center of Soil and Environmental Resources, Agronomic Institute of Campinas (IAC), Campinas, Sao Paulo, Brazil

**Keywords:** Nutrient uptake, Plant biomass, Phosphorus solubilizer, Plant growth-promoting bacteria, Strigolactone, Root architecture

## Abstract

Sorghum is a multipurpose crop that is cultivated worldwide. Plant growth-promoting bacteria (PGPB) have important roles in enhancing sorghum biomass and nutrient uptake and suppressing plant pathogens. The aim of this research was to test the effects of the endophytic bacterial species *Kosakonia radicincitans* strain IAC/BECa 99, *Enterobacter asburiae* strain IAC/BECa 128, *Pseudomonas fluorescens* strain IAC/BECa 141, *Burkholderia tropica* strain IAC/BECa 135 and *Herbaspirillum frisingense* strain IAC/BECa 152 on the growth and root architecture of four sorghum cultivars (SRN-39, Shanqui-Red, BRS330, BRS509), with different uses and strigolactone profiles. We hypothesized that the different bacterial species would trigger different growth plant responses in different sorghum cultivars. *Burkholderia tropica* and *H. frisingense* significantly increased the plant biomass of cultivars SRN-39 and BRS330. Moreover, cultivar BRS330 inoculated with either strain displayed isolates significant decrease in average root diameter. This study shows that *Burkholderia tropica* strain IAC/BECa 135 and *H. frisingense* strain IAC/BECa 152 are promising PGPB strains for use as inocula for sustainable sorghum cultivation.

## Introduction

The plant sorghum (*Sorghum bicolor*) originated on the African continent and is cultivated worldwide (Rao et al., 2014). Sorghum has a short growth period, and is therefore a preferred cereal in arid and semi-arid regions (Farre & Faci, 2006; Wu et al., 2010; Funnell-Harris, Sattler & Pedersen, 2013). In Africa, sorghum is mainly cultivated by small farmers as staple food and for beverage production (Haiyambo, Reinhold-Hurek & Chimwamurombe, 2015). By contrast, sorghum is mostly used for the feed market in North and Central America and for animal feedstock, ethanol production, and soil coverage in South America (Dutra et al., 2013; Perazzo et al., 2013; Damasceno, Schaffert & Dweikat, 2014; Rao et al., 2014). Sorghum is currently the fifth most cultivated cereal worldwide (Ramu et al., 2013) and in 2014, approximately 71 million tons of sorghum grains were produced around the world (FAO, 2018).

Sorghum producers often face yield problems due to soil nutrient deficits, limited access to chemical fertilizers, and the frequent need to combat plant pathogens (Haiyambo, Reinhold-Hurek & Chimwamurombe, 2015). Although conventional agricultural methods, such as chemical fertilization and pesticide application, can be used to overcome these limitations, the environmental side effects of these practices may be unsustainable As an alternative, the use of plant growth-promoting bacteria (PGPB) as biofertilizers not only enhances plant biomass and nutrient uptake but also improves pathogen control (Bhattacharyya & Jha, 2012; Dawwam et al., 2013). PGPB can alter the root architecture and promote plant growth by directly facilitating nutrient acquisition or modulating plant hormone levels or by indirectly inhibiting pathogenic organisms (Bhattacharyya & Jha, 2012; Glick, 2012). The best-known processes of plant nutrient acquisition mediated by PGPB are nitrogen fixation, phosphate (P) solubilization and iron sequestration (Lucy, Reed & Glick, 2004). Different groups of bacteria produce plant growth regulators, such as cytokinins, gibberellins, indole-3-acetic acid (IAA), and ethylene, that may also affect the plant’s hormonal balance (Amara, Khalid & Hayat, 2015). Moreover, PGPB can promote plant growth by fixing N_2_ and inhibiting plant pathogens by producing antibiotics or lytic enzymes or competing for resources, which can limit disease incidence and severity (Glick, 2012; Da Silveira et al., 2016). In addition to the bacterial modification of plant metabolism, plant exudates have the potential to modify rhizosphere microbial community assembly and interactions (Haichar et al., 2014; Vurukonda et al., 2016). Recent studies suggest that the plant hormone strigolactone (SL) plays an important role in plant rhizosphere bacterial community composition (Funnell-Harris, Pedersen & Marx, 2008; Schlemper et al., 2017). Furthermore, Peláez-Vico et al. (2016) showed that the PGPB *Sinorhizobium meliloti* reduces orobanchol and orobanchyl acetate levels in nodulated alfalfa plants under P starvation, suggesting a role of SL in rhizobial–legume interactions. In a *in vitro* assay, Peláez-Vico et al. (2016) demonstrated that swarming motility of *Sinorhizobium meliloti* is triggered by the synthetic SL analogue GR24.

Many aspects of the interaction between PGPB and plants have been addressed for a wide range of plant species. Specifically in sorghum, some studies have focused on the interaction of PGPB strains isolated from third-party host species as inoculants for the sorghum rhizosphere (Matiru & Dakora, 2004; Dos Santos et al., 2017), whereas other works have reported the inoculation in other plant species of bacterial strains isolated from sorghum.

Matching beneficial bacteria with their preferred crops might optimize root colonization and biocontrol (Raaijmakers & Weller, 2001), especially when different plants are cropped in soils with the same bacterial composition. In this context, it is extremely important to identify bacterial candidates that have similar growth effects on plants that share the same soil. In Brazil, sorghum have been planted during the sugarcane off season as well as in former sugarcane fields (May et al., 2013), and therefore sorghum and sugarcane are frequently exposed to the same soil. Endophytic bacteria with plant growth-promoting traits isolated from sugarcane have been shown to increase biomass and plant N content when inoculated in plantlets of sugarcane. For example, Govindarajan et al. (2006) observed an increase in sugarcane yield of 20%, while Sevilla et al. (2001) observed increases of 31% in plant dry matter, 43% in N accumulation, and 25% in productivity in two sugarcane varieties. We hypothesized that different bacterial species isolated from sugarcane will trigger different growth plant responses in different sorghum cultivars. Thus, to determine if endophytic strains characterized as PGPB in sugarcane can act as non-host-specific PGPB benefiting sorghum performance, we tested the effect of five bacterial strains on the plant biomass and root architecture of four *Sorghum bicolor* cultivars with different uses and characteristics: SRN-39, an African grain cultivar that produces high amounts of orobanchol; Shanqui-Red (SQR), a Chinese cultivar that produces high amount of 5-deoxystrigol; BRS330, a hybrid grain cultivar from Brazil and BRS509, a hybrid saccharin cultivar from Brazil that produces both orobanchol and sorgomol (Schlemper et al., 2017).

Inoculation of the cultivars SRN-39 and BRS330 with *Burkholderia tropica* or *Herbaspirillum frisingense* strains resulted in significant increases in plant biomass. Moreover, cultivar BRS330 exhibited significant increases in average root diameter when inoculated with either strain. This study shows that *Burkholderia tropica* strain IAC/BECa 135 and *H. frisingense* strain IAC/BECa 152 are promising PGPB strains for use as inocula for sustainable sorghum cultivation.

## Materials and Methods

### Bacterial isolates and screening of plant growth promotion traits

Five bacterial endophytic strains isolated from sugarcane stem belonging to Agronomic Institute of Campinas (IAC)—Brazil culture collection were used for this experiment: *Kosakonia radicincitans* strain IAC/BECa-99 (KF542909.1), *Enterobacter asburiae* strain IAC/BECa-128 (JX155407.1), *Pseudomonas fluorescens* strain IAC/BECa-141 (KJ588202.1), *Burkholderia tropica* strain IAC/BECa-135 (KJ670083.1), and *H. frisingense* strain IAC/BECa-152 (JX155400.1).

Phosphate solubilization test: the strains were cultured on culture medium containing inorganic phosphate (CaHPO_4_) according to the method of Katznelson & Bose (1959). The experiment was performed in triplicate for 5 days. The ability of the bacteria to solubilize calcium phosphate was verified by the formation of a clear halo surrounding the colonies.

Indole-3-acetic acid test: the strains were grown in culture medium containing L-tryptophan, the precursor of IAA (Bric, Bostock & Silverstone, 1991), covered with a nitrocellulose membrane, and incubated at 28 °C in the dark for 24 h. The nitrocellulose membranes were immersed in Salkowski’s solution and incubated at room temperature for up to 3 h. The formation of a red-purplish halo around the colonies indicated IAA production.

Siderophore production: siderophore production by the strains was measured using the method of Schwyn & Neilands (1987), in which a dye, chromeazurol S (CAS), is released from a dye-iron complex when a ligand sequesters the iron complex. This release causes a color change from blue to yellow–orange. In this case, the ligant was one or more of the siderophores found in the culture supernatants of the bacterial strains.

Hydrogen cyanide (HCN) test: the production of HCN by all strains was assessed according to Bakker & Schippers (1987). Moistened filter paper with picric acid solution (5%) and Na_2_CO_3_ (2%) was added to the top of the Petri dishes and incubated at 28 °C for 36 h. The experiments were performed in triplicate for each strain, and a colour change of the paper from yellow to orange–red indicated the ability to produce HCN.

Antifungal activity assay by paired culture (PC) test: the antagonistic properties of the strains against two pathogenic fungi, *Bipolaris sacchari* and *Ceratocystis paradoxa*, were evaluated using the PC method. The fungi were cultured in Petri dishes containing PDA medium for 7 days (*C. paradoxa*) and 10 days (*Bipolaris sacchari*). For the PC tests, the bacterial strains (grown in DYGS liquid medium for 4 days) were arranged as streaks on one side of the Petri dish with PDA medium; 5 mm of PDA culture medium containing the pathogen mycelial disc was placed on the opposite side. The control treatment contained only the pathogen mycelial disc of the pathogen. An antagonistic reaction was confirmed by the formation of a zone of inhibition (haloes without mycelial growth). The tests were performed using five replicates per treatment.

### Sorghum cultivars

Four sorghum cultivars differing in use, origin, and strigolactone production were chosen for inoculation with the selected PGPB. The cultivars were SRN-39, an African sorghum that produces a high amount of orobanchol; SQR a Chinese sorghum that produces mostly 5-deoxystrigol; BRS330, a hybrid *Sorghum bicolor* grain from Brazil; and BRS509, a hybrid *Sorghum bicolor* saccharin from Brazil that produces both 5-deoxystrigol and sorgomol.

### Mesocosm experiment

**Figure 1:**
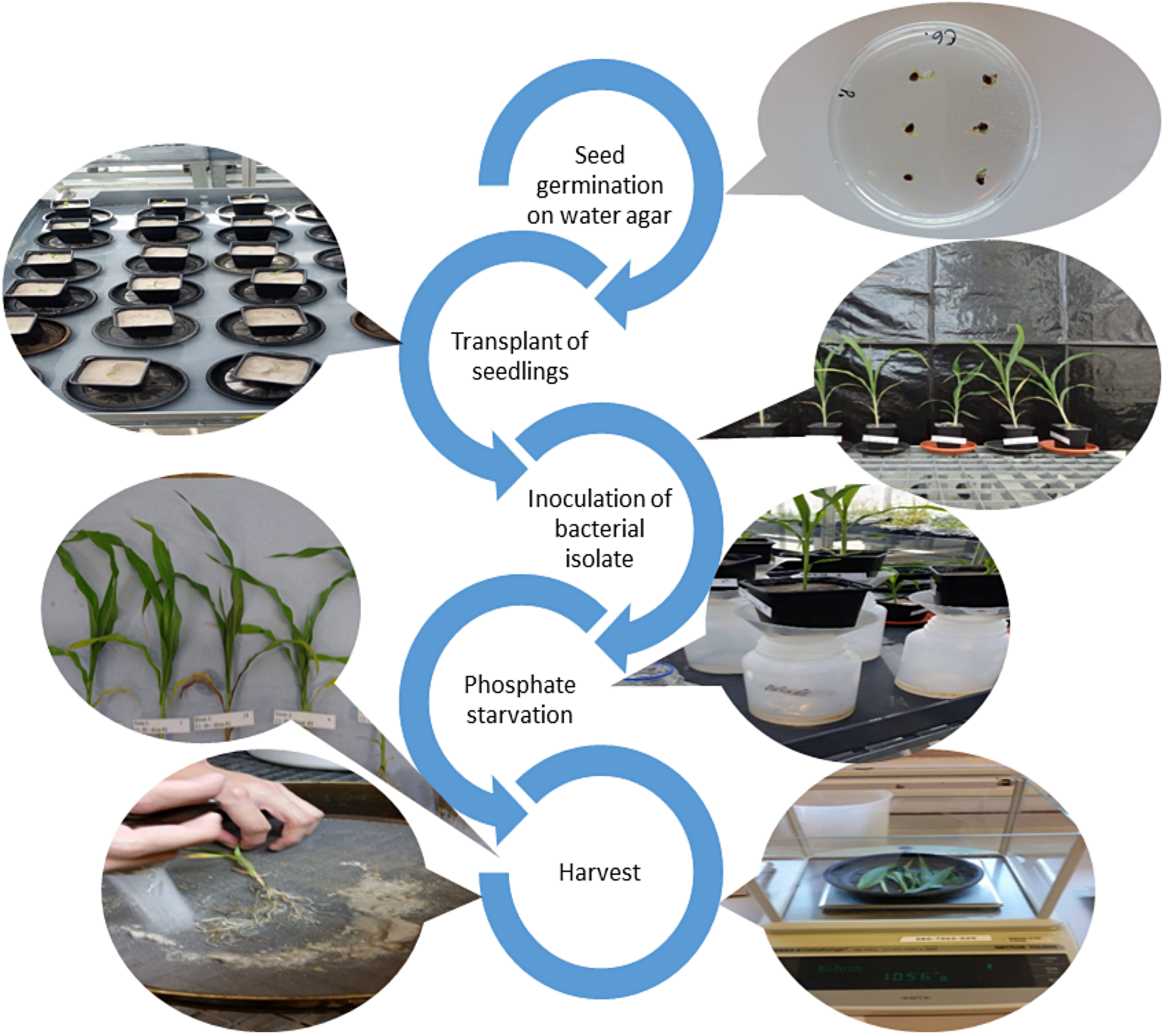
Illustration of the different steps of the study (photos: T. Schlemper and F. Silva Gutierrez).

The experiment was performed in a greenhouse of the Netherlands Institute of Ecology (NIOO-KNAW), located in Wageningen, the Netherlands. The experiment was carried out from September to October 2016, with a total duration of 30 days. A complete random design was used. The treatments consisted of four sorghum cultivars, each one inoculated independently with five bacterial isolates, with a total of nine replicates per treatment. A non-inoculated treatment under phosphate starvation conditions was established as a control. Seeds were disinfected as described by Liu et al. (2013). Briefly, seeds were soaked in 70% ethanol for 3 min, transferred to a new tube containing 2.5% sodium hypochlorite solution, and shaken for 5 min. The seeds were then washed with 70% ethanol solution for 30 s. Finally, the seeds were rinsed with sterile water four times. After the last washing step, 20 μl of the remaining water was plated on Petri dishes with Luria-Bertani (LB) medium to confirm the success of disinfection. After disinfection, the seeds were placed in Petri dishes containing 1% water agar medium, and the plates were incubated at 25 °C for 2 days in the dark for seed germination. The experiment is illustrated in Fig. 1. When radicle emerged from the seed coat, the seedlings were transplanted from the Petri dishes to 11 × 11 × 12 cm plastic pots filled with autoclaved silver sand as substrate. The pots containing one plant each were maintained under greenhouse conditions for 4 weeks. During the first week, the pots were watered with 1/2 Hoagland 10% P nutrient solution, followed by P starvation. To create P starvation conditions, the substrate with plants was first flushed with 500 mL of 1/2-strength Hoagland nutrient solution without phosphate to remove any remaining phosphate in the substrate by drainage through the pot. After 2 days, to simulate field conditions, two g (125 μM) of insoluble tricalcium phosphate (Ca_3_(PO_4_)_2_), which can be solubilized by microorganisms but not taken up directly by the plant (Estrada et al., 2013), was diluted in Hoagland nutrient solution and applied to the pots. The watering regime was maintained by applying 25 ml of nutrient solution every 2 days.

### Bacterial inoculation

Bacterial isolates were taken from single colonies grown in Petri dishes containing LB medium at 30 °C for 2–3 days and stored at 4 °C. Bacterial cells of each strain were then grown overnight at 31 °C in LB liquid medium and subsequently inoculated again in a fresh LB medium until reaching the desired inoculum density (10^8^ cfu ml^−1^) (Mishra et al., 2016). After transplanting, and during plant growth, bacterial isolates were applied three times on the top of the sandy substrate directly at the location of the seedling roots. The control treatment was inoculation with LB medium without bacteria. The first inoculation was performed on the third day after transplanting, the second on the second day after P starvation, and the last 1 week later. The inoculation was performed three times to ensure a sufficient bacterial cell density surrounding the plant roots. Loss or dilution of the bacterial inoculum during either the P starvation treatment or the watering regime was possible due to the great drainage potential of the sandy substrate. A density of 10^8^ cfu ml^−1^ in a volume of 1 ml was used for each bacterial strain at each inoculation time.

### Harvesting

After 4 weeks of transplanting, the experimental plants were harvested, and six plants per treatment were taken for biomass and root architecture measurements. The plants were carefully collected from the pots and the root system was rinsed with tap water to remove sand particles. The plants were then divided into shoot and root parts for root architecture and plant dry biomass measurements.

#### Root architecture

For root architecture measurements, the roots were sectioned in three parts, spread along a rectangular acrylic tray and placed in an EPSON scanner Ver. 3.9.3 1NL. The measured root architecture parameters were the specific root area (SRA), specific root length (SRL), AvD, and specific root density (SRD). All parameters were analyzed in WINRHIZO^™^ program V2005b. Specific root area was calculated by dividing the surface area by the root dry biomass. Specific root length was calculated by the following formula:

$${\rm{SRL\, =\ }}{{{{\displaystyle{\rm{Root\ length}}} / {\displaystyle100}}} \over {{\rm{Root}}\ {\rm{dry}}\ {\rm{biomass}}}} \times 10$$

#### Plant biomass

The shoot and root parts were dried at room temperature for 4 h, until no remaining water could be observed on their surfaces. The fresh weights of both parts were obtained using an electronic scale. The shoot and root parts were then placed in an oven at 60 °C for 72 h. The percentage of biomass was calculated by dividing the dry weight biomass by the fresh weight and multiplying by 100.

### Statistical analysis

The plant dry biomass and root architecture data were analysed by analysis of variance (ANOVA) and Duncan’s test (*P* < 0.05) using the IBM SPSS Statistics 23 program.

## Results

### Bacterial strains and PGPB effect on sorghum plant biomass

The five bacterial strains exhibited different characteristics in terms of specific plant growth-promotion traits (Table 1). Strain IAC/BECa 128 (*E. asburiae*) had the capability to solubilize phosphate. All strains produced IAA, except strain IAC/BECa 135 (*Burkholderia tropica*). Strains IAC/BECa 99 (*K. radicincitans*), IAC/BECa 128 (*E. asburiae*) and IAC/BECa 152 (*H. frisingense*) produced siderophores. Strain IAC/BECa 141 (*P. fluorescens*) produced hydrogen cyanide and strain IAC/BECa 99 (*K. radicincitans*) exhibited amplification of *nif*H gene. All strains were antagonistic to the growth of the pathogens *Fusarium verticillioides* and *C. paradoxa*; however, only strain IAC/BECa 141 (*P. fluorescens*) was antagonistic to the pathogen *Bipolaris sacchari*.

**Table 1:** Plant growth promotion characteristics of five bacterial strains.

PGPR trait	Strain				
	IAC/BECa 99	IAC/BECa 128	IAC/BECa 135	IAC/BECa 141	IAC/BECa 152
P solubilization	−	+	−	−	−
IAA	+	+	−	+	+
Siderophore	+	+	−	−	+
Hydrogen cyanide	−	−	−	+	−
*nifH* gene	+	−	−	−	−
Antagonism to:					
*Bipolaris Sacchari*	−	−	−	+	+
*Fusarium verticillioides*	+	+	+	+	+
*Ceratocystis paradoxa*	+	+	+	+	+

**Notes:**

Plant growth promotion characteristics of five bacterial strains IAC/BECa 99 (*Kosakonia radicincitans*), IAC/BECa 128 (*Enterobacter asburiae*), IAC/BECa 135 (*Burkholderia tropica*), IAC/BECa 141 (*Pseudomonas fluorescens*), and IAC/BECa 152 (*Herbaspirillum frisingense*).

Positive (+) and negative (−) signals mean positive and negative result for each plant growth promotion traits listed.

Sorghum cultivar SRN39 exhibited a significant increase in root dry biomass when inoculated with *Burkholderia tropica* strain IAC/BECa 135 or *H. frisingense* strain IAC/BECa 152, and a significantly higher shoot biomass when inoculated with *E. asburiae* strain IAC/BECa 128 or *H. frisingense* strain IAC/BECa 152, compared with the control (Table 2). Cultivar BRS330 displayed a significant increase in root dry biomass when inoculated with strain IAC/BECa 135 (*Burkholderia tropica*) or IAC/BECa 152 (*H. frisingense*) compared with the control. However, when inoculated with *E. asburiae* strain IAC/BECa 128, this cultivar exhibited a significant decrease in shoot biomass compared with the non-inoculated control. Cultivars SQR and BRS509 did not exhibit any significant differences in biomass when inoculated with any of the strains compared to the control (Table 2).

**Table 2:** Root and shoot biomass (%) of four cultivars of sorghum (SRN-39, SQR, BRS330 and BRS509) inoculated with five bacterial isolates.

Cultivars	Bacterial isolate	Root biomass (%)	Shoot biomass (%)
SRN-39	Control	18.10 ± 1.19^c^	22.20 ± 0.73^c^
	IAC/BECa 99	21.52 ± 0.75^b,c^	23.66 ± 0.50^b,c^
	IAC/BECa 128	24.83 ± 3.03^a,b,c^	25.22 ± 0.98^b^
	IAC/BECa 135	27.48 ± 3.20^a,b^	23.82 ± 0.37^b,c^
	IAC/BECa 141	21.46 ± 2.06^b,c^	23.94 ± 0.68^b,c^
	IAC/BECa 152	31.47 ± 1.74^a^	28.51 ± 1.34^a^
SQR	Control	29.52 ± 2.84^a^	22.21 ± 0.88^a^
	IAC/BECa 99	24.50 ± 2.25^a^	19.62 ± 1.15^a^
	IAC/BECa 128	25.67 ± 1.90^a^	19.56 ± 0.97^a^
	IAC/BECa 135	31.15 ± 3.24^a^	19.69 ± 0.64^a^
	IAC/BECa 141	29.26 ± 3.56^a^	20.31 ± 0.87^a^
	IAC/BECa 152	33.64 ± 1.59^a^	20.94 ± 0.37^a^
BRS330	Control	13.19 ± 0.69^b,c^	20.75 ± 0.35^a^
	IAC/BECa 99	12.58 ± 0.48^b,c^	19.92 ± 0.54^ab^
	IAC/BECa 128	11.77 ± 0.69^c^	19.82 ± 0.32^b^
	IAC/BECa 135	19.17 ± 2.30^a^	21.24 ± 0.33^a^
	IAC/BECa 141	16.14 ± 1.02^a,b^	19.91 ± 0.48^a,b^
	IAC/BECa 152	18.43 ± 0.98^a^	20.50 ± 0.30^a,b^
BRS509	Control	24.13 ± 2.00^a,b^	25.38 ± 1.46^a^
	IAC/BECa 99	20.79 ± 2.60^b^	23.56 ± 0.23^a^
	IAC/BECa 128	24.57 ± 0.88^a,b^	22.73 ± 0.65^a^
	IAC/BECa 135	28.29 ± 2.38^a^	22.48 ± 0.44^a^
	IAC/BECa 141	20.68 ± 1.62^b^	22.97 ± 0.48^a^
	IAC/BECa 152	24.04 ± 1.70^a,b^	23.44 ± 1.50^a^

**Notes:**

Root and shoot biomass (%) of four cultivars of sorghum (SRN-39, SQR, BRS330 and BRS509) inoculated with five bacterial isolates IAC/BECa 99 (*Kosakonia radicincitans*), IAC/BECa 128 (*Enterobacter asburiae*), IAC/BECa 135 (*Burkholderia tropica*), IAC/BECa 141 (*Pseudomonas fluorescens*), and IAC/BECa 152 (*Herbaspirillum frisingense*).

The values are means of replicates (*n* = 6) ± (SE). For each parameter, letters compare (on column) the means between the bacterial inoculums treatments within the same cultivar. Means followed by the same letter are not statistically different by Duncan test (*P* < 0.05).

### PGPB effects on root architecture

Cultivars SRN39, SQR, and BRS509 did not display significant differences in root architecture parameters when inoculated with any strain compared with the control. However, cultivar BRS330 inoculated with *E. asburiae* strain IAC/BECa 128 exhibited a significantly higher SRA and SRL compared with the control. Furthermore, when inoculated with *Burkholderia tropica* strain IAC/BECa 135 or *H. frisingense* strain IAC/BECa 152, the same cultivar exhibited a significant decrease in AvD compared with the control (Table 3). Cultivar BRS 330 inoculated with *Burkholderia tropica* (IAC/BECa 135), *P. fluorescens* (IAC/BECa 141) or *H. frisingense* (IAC/BECa 152) had a higher SRD compared than the treatment inoculated with *E. asburiae* (IAC/BECa 128) but not the control.

**Table 3:** Specific root area, specific root length, average of root diameter, and specific root density of our cultivars of sorghum.

Cultivars	Isolates	SRA (cm^2^/g)	SRL (cm/g)	AvD (mm)	RDENS (cm^3^/g)
SRN-39	Control	847.29 ± 44.46^a^	687.39 ± 66.85^a^	0.40 ± 0.02^a^	0.12 ± 0.00^a^
	IAC/BECa 99	1027.44 ± 130.77^a^	867.21 ± 151.8^a^	0.39 ± 0.02^a^	0.11 ± 0.01^a^
	IAC/BECa 128	1061.95 ± 146.42^a^	881.21 ± 66.73^a^	0.38 ± 0.03^a^	0.11 ± 0.02^a^
	IAC/BECa 135	1016.59 ± 59.11^a^	882.89 ± 91.54^a^	0.38 ± 0.02^a^	0.11 ± 0.01^a^
	IAC/BECa 141	1044.78 ± 68.45^a^	883.43 ± 67.29^a^	0.38 ± 0.02^a^	0.10 ± 0.01^a^
	IAC/BECa 152	914.74 ± 50.78^a^	802.62 ± 69.77^a^	0.37 ± 0.01^a^	0.12 ± 0.00^a^
SQR	Control	1021.64 ± 41.81^a^	909.61 ± 69.28^a^	0.36 ± 0.01^a^	0.11 ± 0.00^a,b^
	IAC/BECa 99	1034.82 ± 58.91^a^	922.61 ± 62.72^a^	0.36 ± 0.01^a^	0.11 ± 0.01^a,b^
	IAC/BECa 128	1217.28 ± 204.39^a^	1220.4 ± 310.4^a^	0.35 ± 0.02^a^	0.10 ± 0.01^a,b^
	IAC/BECa 135	1239.66 ± 109.67^a^	1056.5 ±116.53^a^	0.38 ± 0.02^a^	0.09 ± 0.01^b^
	IAC/BECa 141	1284.28 ± 207.40^a^	1214.2 ± 211.88^a^	0.34 ± 0.01^a^	0.10 ± 0.01^b^
	IAC/BECa 152	917.84 ± 26.31^a^	894.24 ± 32.09^a^	0.33 ± 0.01^a^	0.13 ± 0.00^a^
BRS330	Control	881.00 ± 28.55^b^	609.24 ± 23.55^b^	0.46 ± 0.01^a^	0.10 ± 0.00^a,b^
	IAC/BECa 99	926.69 ± 66.26^a,b^	677.58 ± 47.12^a,b^	0.43 ± 0.01^a,b^	0.10 ± 0.01^a,b^
	IAC/BECa 128	1101.94 ± 96.50^a^	774.88 ± 72.32^a^	0.46 ± 0.01^a^	0.08 ± 0.01^b^
	IAC/BECa 135	841.07 ± 80.40^b^	645.94 ± 60.04^a,b^	0.41 ± 0.01^b^	0.12 ± 0.01^a^
	IAC/BECa 141	767.82 ± 44.72^b^	561.31 ± 32.04^b^	0.44 ± 0.01^a,b^	0.12 ± 0.01^a^
	IAC/BECa 152	786.94 ± 14.98^b^	605.28 ± 18.08^b^	0.42 ± 0.01^b^	0.12 ± 0.00^a^
BRS509	Control	1337.8 ± 665.09^a^	1121.9 ± 531.2^a^	0.38 ± 0.03^a,b^	0.17 ± 0.04^a^
	IAC/BECa 99	707.81 ± 68.59^a^	553.35 ± 62.87^a^	0.41 ± 0.01^a^	0.14 ± 0.01^a^
	IAC/BECa 128	633.89 ± 45.40^a^	524.96 ± 35.46^a^	0.38 ± 0.01^a,b^	0.17 ± 0.01^a^
	IAC/BECa 135	779.74 ± 53.88^a^	662.51 ± 50.39^a^	0.38 ± 0.01^a,b^	0.14 ± 0.01^a^
	IAC/BECa 141	1130.4 ± 230.66^a^	1138 ± 325.22^a^	0.34 ± 0.02^b^	0.12 ± 0.01^a^
	IAC/BECa 152	703.37 ± 79.40^a^	622.32 ± 74.82^a^	0.36 ± 0.01^a,b^	0.17 ± 0.02^a^

**Notes:**

Specific root area (SRA), specific root length (SRL), average of root diameter (AvD), and specific root density (RDENS) four cultivars of sorghum (SRN-39, SQR, BRS330 and BRS509) inoculated with five bacterial isolates (IAC/BECa 99 (*Kosakonia radicincitans*), IAC/BECa 128 (*Enterobacter asburiae*), IAC/BECa 135 (*Burkholderia tropica*), IAC/BECa 141 (*Pseudomonas fluorescens*), and IAC/BECa 152 (*Herbaspirillum frisingense*).

Values are means of replicates (*n* = 6) ± (SE). For each parameter, letters compare (on column) the means between the bacterial inoculum treatments within the same cultivar. Means followed by the same letter are not statistically different by Duncan test (*P* < 0.05).

## Discussion

This work aimed to evaluate the effect of five bacterial strains isolated from sugarcane on the plant growth and root architecture of four sorghum cultivars. Cultivars SRN-39 and BRS330 inoculated with *Burkholderia tropica* strain IAC/BECa 135 or *H. frisingense* strain IAC/BECa 152 and cultivar SRN-39 inoculated with *E. asburiae* strain IAC/BECa 128 or *H. frisingense* strain IAC/BECa 152 exhibited significant increases in sorghum root and shoot biomass, respectively, compared with the control. Although the number of replicates in our study was small (6) our results corroborate those of Chiarini et al. (1998), who found that isolates belonging to the genera *Burkholderia* and *Enterobacter* co-inoculated in the sorghum rhizosphere promoted a significant increase in root growth compared to non-inoculated plants. Furthermore, species belonging to the genera *Burkholderia* and *Herbaspirillum* promote the growth of sugarcane and maize (Pereira et al., 2014; Da Silva et al., 2016), which, like sorghum, are C4 grass species. *H. frisingense* strain IAC/BECa 152 produces siderophores and IAA, whereas *Burkholderia tropica* strain IAC/BECa 135 does not. The strain IAC/BECa 152 might possess a set of mechanisms that improve plant nutrient uptake either by increasing nutrient availability in the rhizosphere or influencing the biochemical mechanisms underlying nutritional processes (Pii et al., 2015). Such mechanisms include changes in the root system architecture and shoot-to-root biomass ratio, increases in proton efflux by modulating H^+^ATPase activities, indirect effects of IAA produced by PGPB, or acidification of the rhizosphere to enhance nutrient solubility (Pii et al., 2015). In addition to growth regulators, siderophores can be produced and are known to assist Fe acquisition by roots (Saravanan, Madhaiyan & Thangaraju, 2007; Mehnaz et al., 2013).

In contrast to the effects of *Burkholderia tropica*, *H. frisingense* and *E. asburiae* on the growth of both grain sorghum cultivars, these strains had no significant effect on BRS 509 (sweet sorghum) and on SQR cultivars compared with the control. Interestingly, in accordance with our results, Dos Santos et al. (2017) observed significant increase in the biomass of grass and grain sorghum inoculated with *Burkholderia* ssp. or *Herbaspirillum* ssp. but not sweet sorghum inoculated with the same isolates. Taken together with our results, these findings suggest that the effects of strains of *Burkholderia tropica* and *H. frisingense* on plant growth are dependent on sorghum genotype. It is unclear why the effects of these strains were greater in certain sorghum cultivars than others. However, different sorghum genotypes release different SL molecules in different quantities under P starvation (Schlemper et al., 2017). Thus, the high relative abundance of the genus *Burkholderia* in the rhizosphere of sorghum cultivar SRN-39 could be related to the level of orobanchol, which is 300 and 1,100 times higher in SRN-39 than in the cultivars SQR, BRS330 and BRS509 as suggested by Schlemper et al. (2017).

When inoculated with *E. asburiae* strain IAC/BECa 128, cultivar BRS330 displayed an increase in SRL and area compared with the control. These finding are in agreement with a study by Kryuchkova et al. (2014), who reported that *Enterobacter* species can promote increased root length and lateral roots in sunflower. The strain IAC/BECa 128 can solubilize phosphate, which trait might explain the increase in root biomass. The cultivar BRS330 inoculated with strain IAC/BECa 135 (*Burkholderia tropica*) or IAC/BECa 152 (*H. frisingense*) exhibited a significant decrease in average root diameter compared with the control but slight increase in root density. Plants under P deficiency conditions may increase root density probably to enhance nutrient acquisition (Kapulnik & Koltai, 2014). Moreover, species belonging to the genus *Herbaspirillum* can influence plant root architecture and improve signaling pathways of plant hormone production (Straub et al., 2013). No significant effects on sorghum growth or root architecture modification were observed when *K. radicincitans* strain IAC/BECa-99 or *P. fluorescens* strain IAC/BECa-141 was inoculated in the rhizosphere of any evaluated sorghum cultivar. Although there are many reports on the effects of *K. radicincitans* (formerly known as *E. radicincitans*) on a range of plants, such as *Arabidopsis thaliana,* radish, and tomato (Berger, Brock & Ruppel, 2013; Brock et al., 2013; Berger et al., 2015), there are no reports on the effects of this bacterial species on sorghum growth or root architecture modification. With respect to *P. fluorescens,* Marcos et al. (2016) found that the strain IAC/BECa 141, when used as an inoculant applied to two sugarcane varieties, increased chlorophyll *a* content without changing plant growth. Kumar et al. (2012) studying the effect of seven different fluorescent *Pseudomonas* spp. strains with single or multiple PGPR traits, in sorghum growth, observed that all strains were able to increase sorghum growth compared to a non-inoculated control. Our results demonstrated that selected bacterial strains characterized as PGPB in sugarcane were able to promote plant growth and root architecture modification in sorghum. Based on the reproducibility of the performance of bacterial strains for different crops, our findings shed light on the identification of bacterial candidate strains for improving the growth and yield of crops that share the same soil bacterial source in intercropping or crop rotation systems. However, since we did not evaluate the bacterial community that actually colonized the root system, it is difficult to confirm that the differences we observed in sorghum biomass were due to the effects of the inoculation or a side effect of the plant due to the simple presence of these bacterial cells in the rhizosphere. Hence, we strongly recommend future studies to recover the bacterial community from the endosphere and rhizosphere compartments as proof of inoculation. We suggest that SL plays a role in the effectiveness of PGPB in promoting the growth of specific sorghum genotypes, although our experimental set-up did not allow us to make a straightforward conclusion. More specific experiments are needed to better address the relationship between plant SL production and plant bacterial infection.

## Conclusion

Here, we demonstrated that bacteria strains characterized as PGPB in sugarcane were able to promote plant growth and root architecture modification in sorghum. Our results demonstrated that cultivars SRN-39 and BRS330 inoculated with *Burkholderia tropica* strain IAC/BECa 135 or *H. frisingense frisingense* strain IAC/BECa 152 exhibited a significant increase in plant biomass. Moreover, cultivar BRS330 inoculated with either strain displayed a significant decrease in AvD. The results of this study indicate that *Burkholderia tropica* strain IAC/BECa 135 and *H. frisingense* strain IAC/BECa 152 are promising PGPB strains for use as inocula for sustainable sorghum cultivation.

## Supplemental Information

10.7717/peerj.5346/supp-1 Supplemental Information 1 Plant biomass and root architecture raw data. Sheet 1: Plant biomass raw data.
Sheet 2: Root architecture raw data.

## Additional Information and Declarations

### Data Availability

The following information was supplied regarding data availability: The raw data are provided in the Supplemental File.
